# Establishment of a Survival and Toxic Cellular Model for Parkinson’s Disease from Chicken Mesencephalon

**DOI:** 10.1007/s12640-012-9367-y

**Published:** 2012-12-13

**Authors:** Amparo Tolosa, Xiaolai Zhou, Björn Spittau, Kerstin Krieglstein

**Affiliations:** 1 Department of Molecular Embryology, Institute of Anatomy and Cell Biology, University of Freiburg, Albertstrasse 17, 79104 Freiburg, Germany; 2 Faculty of Biology, University of Freiburg, Freiburg, Germany; 3 FRIAS-Lifenet, University of Freiburg, 79104 Freiburg, Germany

**Keywords:** Dopaminergic neurons, Embryonic chicken midbrain, Neuroprotection, Parkinson’s disease

## Abstract

Cellular models for Parkinson’s disease (PD) represent a fast and efficient tool in the screening for drug candidates and factors involved in the disease pathogenesis. The objective of this study was to establish and characterize a survival and toxic cellular model for PD by culturing dopaminergic neurons from embryonic chicken ventral midbrain. We show that as in rodents, the common neurotrophic factors—brain-derived neurotrophic factor (BDNF), glial cell line-derived neurotrophic factor (GDNF), and fibroblast growth factor 2 (FGF2)—are able to support the survival of chicken midbrain dopaminergic neurons. Furthermore, after treatment with MPP^+^ or rotenone as in vitro models for PD, the number of tyrosine hydroxylase-positive cells decreased drastically. This effect could be significantly rescued by treatment with BDNF or GDNF. Together, our results indicate that mechanisms of neuroprotection of dopaminergic neurons are conserved between chicken and mammals. This supports the use of primary culture from chicken embryonic midbrain as a suitable tool for the study of neuroprotection in vitro.

## Introduction

Parkinson’s disease (PD) is one of the most common neurodegenerative disorders of the central nervous system. It is characterized by chronic and progressive loss of midbrain dopaminergic (DA) neurons, and affects approximately 5.2 million people around the world (Mathers et al. 2008), with consequent social and economic burden. Over the past years, several cellular and animal models have provided insight on the molecular pathways involved in PD. The precise etiology and disease pathogenesis, however, are mostly unknown. Current treatment strategies aim at symptomatic relief rather than preventing disease progression. The use of neurotrophic factors represents a potential therapeutic strategy, not only to increase the survival of the remaining DA neurons but also to support those transplanted (Poewe 2006; Schapira 2009). Within this context, different neurotrophic factors such as neurotrophin, fibroblast growth factor (FGF), epidermal growth factor (EGF), ciliary neurotrophic factor (CNTF), insulin-like growth factor (IGF), transforming growth factor-beta (TGF-β), and interleukin families have been identified to promote survival of DA neurons (von Bohlen und Halbach and Unsicker 2009). Several of these factors have been tested using both, animal and cellular systems, providing evidence that the two approaches are robust and contribute to a better understanding of the disease. Advantages of the cellular models for PD are the rapid screening for drug candidates or factors involved in disease pathogenesis and the consequent decrease in the necessary number of animal experiments.

The chicken model system has been widely used in the field of developmental biology (Stern 2005) and offers many advantages. First, chicken eggs constitute an abundant and non-expensive source of biological material. Second, access to the chicken embryo is easy, providing the possibility to perform temporal and spatial genetic manipulations or directly interfere in protein activity by treatment with specific inhibitors. Also, unlike in rodent models, working with chicken embryos does not involve the death of the mother. Furthermore, the chicken genome possesses similar number of genes compared to humans and it shows a very high level of conserved synteny with mammals (Wallis et al. 2004).

Taking into account the convenience of the chicken model system, our group has previously investigated the role of neurotrophic factors in the development, differentiation, and survival of chicken midbrain dopaminergic neurons in vivo (Farkas et al. 2003). However, there is still no chicken PD cellular model available. In this study, we have established and characterized a cellular PD model by culturing dopaminergic neurons from embryonic chicken ventral midbrain. Using this model, we provide evidence that as in rodents, the common neurotrophic factors brain-derived neurotrophic factor (BDNF), glial cell line-derived neurotrophic factor (GDNF), and fibroblast growth factor 2 (FGF2) are able to support the survival of chicken midbrain dopaminergic neurons. In addition, treatment with the toxins MPP^+^ or rotenone reduced the number of dopaminergic neurons, which could be significantly rescued by treatment with BDNF or GDNF.

## Materials and Methods

### Dissection of Embryonic Chicken Ventral Midbrain

**Fig. 1:**
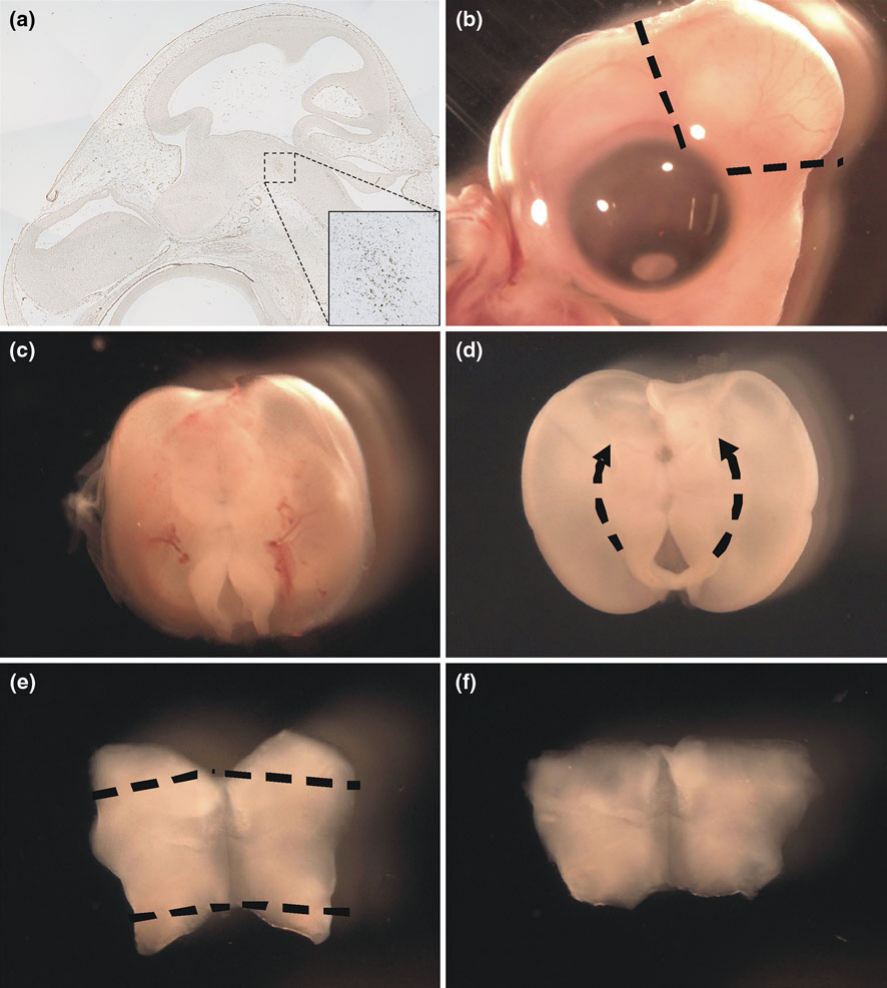
Dissection of the ventral midbrain from embryonic E7 chicken. **a** Sagittal section showing the localization of dopaminergic neurons in midbrain at E7 after immunohistochemistry with anti-tyrosine hydroxylase (TH) antibody. After taking off the amnion membrane, the midbrain (**c**) was dissected from the chicken embryo (**b**). Following the removal of the meninges, the ventral midbrain (**d**) was dissected and trimmed to a “*butterfly*” shape (**e**, **f**)

Fertilized white leghorn eggs (*Gallus gallus*) were incubated in a humidified egg chamber at 38 °C for 7 days. At embryonic day 7 (E7), eggs were removed from the incubator, and cleaned with 70 % ethanol two to three times. Eggshells were then opened and embryos collected and washed in Ca^2+^–Mg^2+^-free Hank’s BSS solution twice (PAA, Cölbe, Germany). The amnion membrane was removed (Fig. 1b) and the midbrain was dissected from the embryo (Fig. 1c). Then, the meninges and vessels were removed and the ventral midbrain (Fig. 1d) was dissected and trimmed to a “butterfly” shape (Fig. 1e, f).

### E7 Chicken Ventral Midbrain Dopaminergic Neurons Culture Preparation

Ventral midbrains were collected and enzymatically dissociated with 750 μl of Trypsin–EDTA (1×) (PAA) for 15 min at 37 °C. An equal amount of ice-cold fetal calf serum (Invitrogen, Germany) with DNase I (at a final concentration of 0.5 mg/ml; Roche Diagnostics, Germany) was added prior to dissociation with wide- and narrow-bored polished Pasteur pipettes. Single cells were washed and collected in serum-free Dulbecco’s modified Eagle’s medium (DMEM)–Ham’s F12 (PAA) medium with N_2_ supplement (Invitrogen), 0.25 % BSA (Sigma, Germany), and antibiotics (penicillin and streptomycin, PAA). Cells were then seeded onto glass coverslips precoated with polyornithine (0.1 mg/ml, Sigma) and laminin (1 μg/ml, Sigma) at a density of 250,000 cells/cm^2^. Cultures were maintained at 37 °C in a humidified 5 % CO_2_ atmosphere.

### Survival-Promoting Activity Test

E7 chicken ventral midbrain cells were treated with or without the following neurotrophic factors: GDNF, BDNF, and FGF2 (5 ng/ml) in the maintenance medium (DMEM/F12 with N_2_ supplement and 0.25 % BSA) from day in vitro (DIV) 1–8. Medium was changed every 2 days. On DIV8, cells were fixed with 4 % paraformaldehyde (PFA) in phosphate buffer (1× PBS, pH 7.4) and used for immunofluorescence or immunocytochemistry. All the growth factors were obtained from Peprotech (Hamburg, Germany).

### MPP^+^- and Rotenone-Induced Toxicity Cellular Model

E7 chicken ventral midbrain cells were cultured in maintenance medium for 5 days with a medium change every 2 days. At DIV5, cells were treated with the neurotoxin MPP^+^ (1 μM) or rotenone (100 nM) with or without GDNF or BDNF (10 ng/ml) for 2 days (DIV5-7). Then, cells were incubated with maintenance medium for one additional day (DIV7–8). At DIV8, the cells were fixed with 4 % PFA and used for immunocytochemistry. MPP^+^ was obtained from Sigma and rotenone from Merck KGaA (Darmstadt, Germany).

### Mink Lung Epithelial Cell Assay

This assay is based on the ability of TGF-β to stimulate plasminogen activator inhibitor-1 (PAI-1) expression (Abe et al. 1994). Mink lung epithelial cells (MLEC), stably transfected with an expression construct containing a truncated PAI-1 promoter fused to the firefly luciferase reporter gene, were incubated overnight with culture medium harvested from either treated or control cells. Latent TGF-β was activated by acidification of the medium as described previously (Gohla et al. 2008). Luciferase activity was quantified in duplicates using a luminometer (Lumat B5076, Berthold, Bad Wildbad, Germany).

### Immunohistochemistry

Whole heads of E7 embryos were immersion fixed in 60 % ethanol, 10 % acetic acid, and 30 % formaldehyde solution at 4 °C overnight. On the following day, the tissue was dehydrated in graded series of ethanol and embedded in paraffin wax. Sections of 10 μm were deparaffinized and heated for 10 min in citrate buffer, pH 6, in a microwave oven to improve antigen retrieval. Monoclonal anti-tyrosine hydroxylase antibody (TH, 1:600, rabbit anti-mouse, Merck Millipore, Darmstadt, Germany) was visualized by the avidin-biotinylated peroxidase method using the Vectastain ABC Kit (Vector Laboratories, USA) and diaminobenzidine (DAB) as chromogen.

### Immunofluorescence and Immunocytochemistry

After fixation with 4 % PFA (15 min), cells were washed with 1× PBS (three times, 3 min each) and incubated with blocking solution containing 10 % normal goat serum (Invitrogen) and 0.1 % Triton X-100 (Roche, Germany) in 1× PBS for 0.5–1 h at room temperature (RT). Thereafter, cells were incubated with primary antibodies for tyrosine hydroxylase (TH, 1:1000, Merck Millipore, Darmstadt, Germany), dopamine beta hydroxylase (DBH, 1:800, Novus Biologicals, Cambridge, UK), neuronal nuclei (NeuN, 1:1000, Millipore), astrocyte marker glial fibrillary acidic protein (GFAP 1:10,000, Sigma), and neuronal progenitor or immature neurons marker vimentin (VIM 1:500, Progen Biotechnik, Heidelberg, Germany) at 4 °C overnight. For immunofluorescence, cells were then incubated with the corresponding Cy3-conjugated secondary antibody (goat anti-mouse Cy3 1:100, goat anti-rabbit Cy3 1:100; Abcam, Cambridge, UK). Nuclei were counterstained using 4′,6-diamidino-2-phenylindole (DAPI 1:1000, Roche). For DAB staining, cells were incubated with anti-rabbit IgG peroxidase conjugate (Sigma) prior to developing with DAB.

The neurotrophic or toxic effects on the dopaminergic neurons were assessed by counting all TH-positive (TH+) cells on a coverslip. For the cellular composition of the culture, NeuN, GFAP, or Vimentin-positive cells were quantified by counting cells in ten randomly selected non-overlapping fields captured at 20X. Then the ratio of positive cells among total cells (DAPI stained) was calculated. All experiments were performed independently at least three times in duplicates. Phase-contrast images were captured using the Leica AF6000 imaging system (LEICA, Wetzlar, Germany), and immunofluorescence and DAB images were captured using Zeiss Axioplan2 microscope (Zeiss, Göttingen, Germany).

Measurements of the cell body, number, and length of processes of the TH+ cells in all conditions were performed with ImageJ software (version 1.44, National Institute of Health, Bethesda, USA) on images captured at 40X magnification. For this, a minimum of 50 cells/coverslip from three different experiments were recorded. Number and length of processes were only considered when longer than the diameter of the cell. In this case, longest processes per cell were measured (1 or 2 per cell).

### Statistical Analysis

The data are expressed as mean ± SEM. Statistical significance between multiple groups was compared by Kruskal–Wallis test for the number of processes and one-way ANOVA for the other variables. When significant differences were found, two-group analysis was performed using Mann–Whitney *U* test for comparing the number of processes and Student’s *t* test for the other comparisons. Values of *p* < 0.05 were considered as statistically significant. All statistical analyses were performed using the GraphPad Prism4 software (GraphPad Software Inc.).

## Results

### Standardizing Primary Culture Derived from E7 Chicken Ventral Mesencephalon

TH in chicken is first detected in ventral midbrain around E7 (Fig. 1a), i.e., within the developmental period (E6–E10), characterized by the stabilization of the dopaminergic phenotype, and the promotion of survival.

**Fig. 2:**
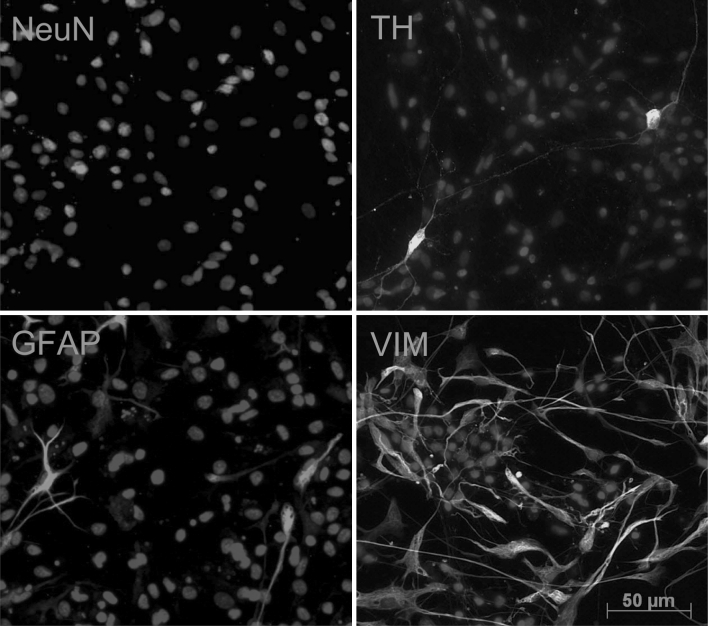
Cellular composition of the chicken ventral midbrain neuron culture at day in vitro (DIV) 7. Cultures were stained for neuronal marker NeuN, 29.1 ± 4.29 %, dopaminergic marker TH, 0.36 ± 0.03 %, astrocytes marker GFAP 7.9 ± 0.68 %, and neuronal progenitor vimentin 40.2 ± 6.02 % and counterstained with DAPI. Percentages of positively stained cells are given as ±SEM, taking the number of DAPI positive nuclei as 100 %. All experiments were performed at least three times

To establish the primary culture, we dissected the ventral midbrain area from E7 chicken embryos as shown in Fig. 1. Resulting tissue was dissociated and cells were plated under serum-free conditions. Fresh serum-free medium was added 24 h after plating, followed by successive changes every 2 days. After 8 days in culture, the analysis of cellular populations revealed 7.9 % of cells from the glial lineage (positive for GFAP), 29.1 % of cells from neuronal lineage (positive for NeuN), 40.2 % of neural precursors (positive for vimentin), and 0.36 % of TH-positive cells (Fig. 2).

We considered TH+ cells in the cell culture as dopaminergic neurons on the basis of two main aspects. First, the dissected area corresponds to the ventral midbrain area where dopaminergic neurons are concentrated (Fig. 1a). Second, double immunostainings of cell culture with DBH (marker of noradrenergic neurons) and TH showed that just 1.45 % cells co-localize DBH in TH+ cells (data not shown). Furthermore, none of the TH+ DBH+ cells had a neuronal morphology, which asserts that the culture was dopaminergic.

### Neurotrophic Factors BDNF, GDNF, and FGF Increase Numbers of TH+ Neurons in Chicken Midbrain Culture in Survival Assay

After establishing a primary culture system derived from chicken ventral midbrain, we addressed the question whether treatment with factors well known for promoting survival of midbrain DA neurons would also have a neurotrophic effect in the chicken system.

We selected GDNF, BDNF, and FGF2 for analyses. These three factors are conserved throughout evolution, with 77.8, 91.5 and 90.8 % of protein identity, respectively, between mice and chicken. They belong to different families of growth factors—therefore, signal via different receptors. In this way, we would test the suitability of the chicken culture survival assay through various mechanisms promoting neuroprotection.

**Fig. 3:**
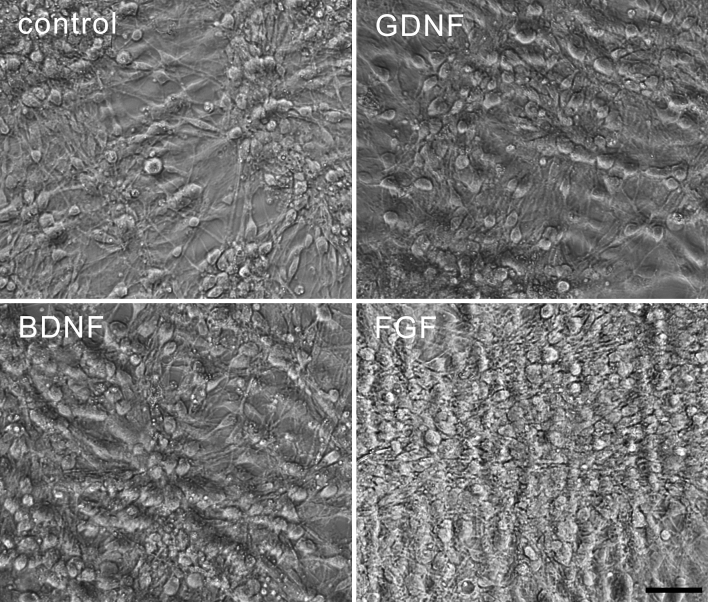
Midbrain dopaminergic neuron culture from E7 chicken. The cultured midbrain dopaminergic neurons were treated with or without classical neurotrophic factors: GDNF, BDNF, and FGF2 (5 ng/ml). Phase-contrast photomicrographs of DIV8 cultures showed that the total number of cells increased after treatment with neurotrophic factors, especially, after FGF2 treatment. All the pictures were taken under the same magnification. *Scale bar* indicates 100 μm

**Fig. 4:**
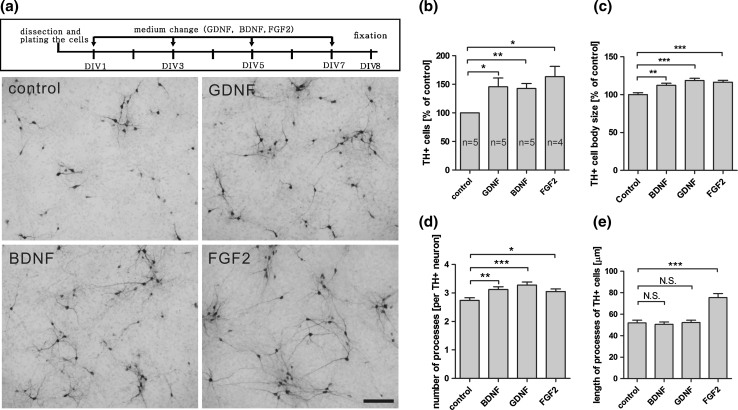
The application of neurotrophic factors on embryonic E7 chicken ventral midbrain cells promotes survival of dopaminergic neurons. Embryonic chicken ventral midbrain dopaminergic neuron cultures were treated with or without the classical neurotrophic factor GDNF, BDNF, and FGF2 (5 ng/ml) from DIV1 to DIV8. **a** Representative pictures show that all three classical neurotrophic factors significantly increased the number of TH+ neurons, **b**–**e** quantification of the number, cell body size, number of processes, and length of processes of the TH+ cells under all conditions. All pictures were taken under the same magnification and the* scale bar* indicates 100 μm. Data from **b** to **e** are presented as mean ± SEM. *p* values derived from Student’s *t* test (number of TH+ cells, cell body size, and length of processes) and Mann–Whitney *U* test (number of processes) are *p < 0.05, **p < 0.01, ***p < 0.001, *NS* not significant. All the experiments were repeated at least three times

A survival assay was performed by treating the chicken midbrain culture with the above neurotrophic factors under serum-free medium conditions at DIV1, DIV3, DIV5, and DIV7. Figure 3 shows phase-contrast photomicrographs of the cells after the treatment. On DIV8, the number of TH–labeled neurons was significantly increased after treatment with GDNF, BDNF or FGF2 when compared to control (Fig. 4), suggesting a neuroprotective effect on the DA neurons consistent with that observed in other species (Beck et al. 1993; Ferrari et al. 1989; Studer et al. 1996; Widmer et al. 2000). Treatment with these factors also increased the size of the cell body and the number of processes in TH-labeled neurons (Fig. 4c, d). In addition, treatment with FGF2 promoted an increase in the length of the neuron processes (Fig. 4e).

**Fig. 5:**
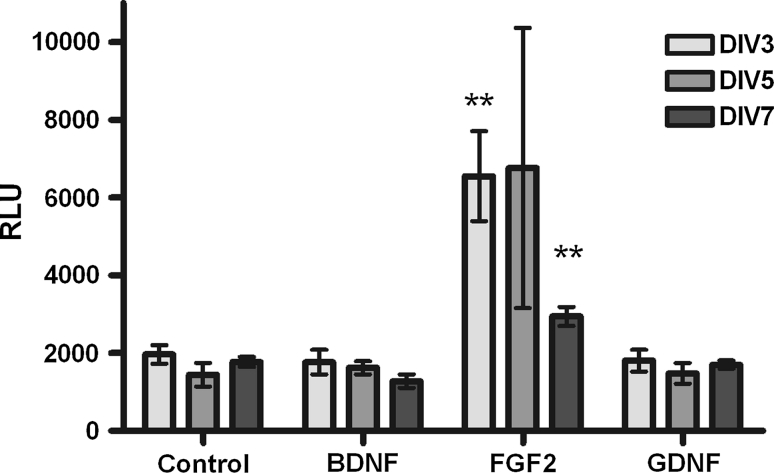
Mink lung epithelial cells–luciferase assay for TGF-β release. MLEC cells were incubated overnight with culture medium harvested from either treated or control cells on DIV3, DIV5, and DIV7. Luciferase activity in relative light units is shown for all conditions. Conditional medium from cells treated with FGF2 showed an increase in TGF-β when compared with the conditional mediums from control, BDNF- and GDNF-treated cells indicating that the mechanism of action for FGF2 is conserved between chicken and mice. Data are presented as mean ± SEM. *p* values derived from Student’s *t* test are **p < 0.01. All the experiments were repeated at least three times

It has been reported that the survival-promoting effect of FGF2 on rat DA neurons is mediated by the neurotrophic factor TGF-β, released from glial cells (Krieglstein et al. 1998). To investigate if the same mechanism exists in chicken DA neurons, TGF-β in the culture medium was determined using a mink lung epithelial cell assay. Culture mediums of control and FGF2-treated cultures were collected on DIV3, DIV5, and DIV7, acidified and tested for TGF-β activity. Treatment with FGF2 increased TGF-β secretion when compared with the conditional mediums from control, BDNF- and GDNF-treated cells (Fig. 5), suggesting that the mechanism of action for FGF2 is conserved between chicken and rodents.

Together, these results indicate that the general mechanisms leading to neuroprotection in midbrain are conserved in chicken when compared with other mammals and support the use of chicken midbrain culture as in vitro model for studying these processes.

### Neurotrophic Factors BDNF and GDNF Protect mDA Chicken Neurons from MPP^+^ and Rotenone in vitro

**Fig. 6:**
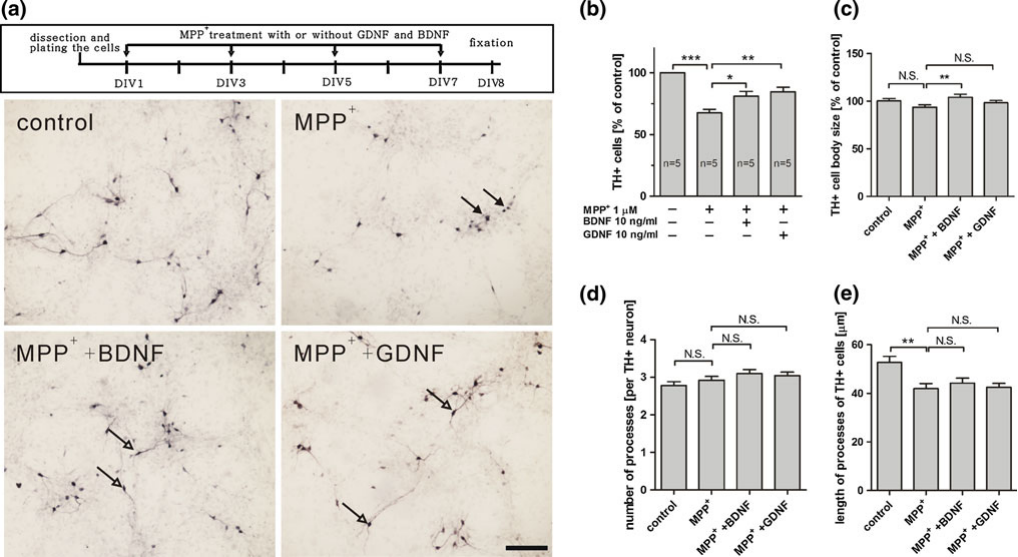
Application of embryonic chicken ventral midbrain culture to evaluate neuroprotection in a MPP^+^-induced toxicity model. Embryonic chicken ventral midbrain cultures were maintained in serum-free conditions from DIV 1 to DIV5 and then treated with MPP^+^ (1 μM) with or without GDNF or BDNF (10 ng/ml) for 2 days (DIV5–7). On DIV8, cells were fixed and stained for TH. **a** Representative pictures of the different treatments show that 1 μm MPP^+^ remarkably reduced the number of TH+ cells, which was significantly rescued by both GDNF and BDNF. Dying and healthy TH+ neurons are depicted by black and white arrows, respectively. **b**–**e** Quantification of the number, cell body size, number of processes, and length of processes of the TH+ cells under all conditions. All pictures were taken under the same magnification and the *scale bar* indicates 100 μm. Data from **b** to **e** are presented as mean ± SEM. *p* values derived from Student’s *t* test (number of TH+ cells, cell body size, and length of processes) and Mann–Whitney *U* test (number of processes) are *p < 0.05, **p < 0.01, ***p < 0.001, *NS* not significant. All the experiments were repeated at least three times

Following analysis of the chicken primary culture from ventral midbrain area for survival assays using neurotrophic factors, we explored its suitability as an in vitro model for Parkinson′s disease. Hence, we first examined the neuroprotective effect of GDNF and BDNF against the specific dopaminergic neurotoxin MPP^+^. The specificity of this molecule is conferred by its high affinity to the dopamine transporter (DAT) present in DA neurons. Once taken up by the cell, MPP^+^ can accumulate in the mitochondria where it inhibits complex I of the electron transport chain leading to mitochondrial intoxication. Primary chicken ventral midbrain culture cells were maintained for 5 days in serum-free conditions before the addition of MPP^+^ (1 μM) for 2 days, with or without BDNF and GDNF. Eight days after plating, the number of TH+ neurons was significantly decreased after MPP^+^ treatment. This neurotoxic effect was significantly reduced by co-treatment with GDNF or BDNF (Fig. 6). Under neurotoxic treatment, all DA neurons showed a decrease in the length of the neuronal processes with no changes in the numbers of processes and the cell body size, but a slight increase in cell body size after BDNF treatment (Fig. 6c–e).

**Fig. 7:**
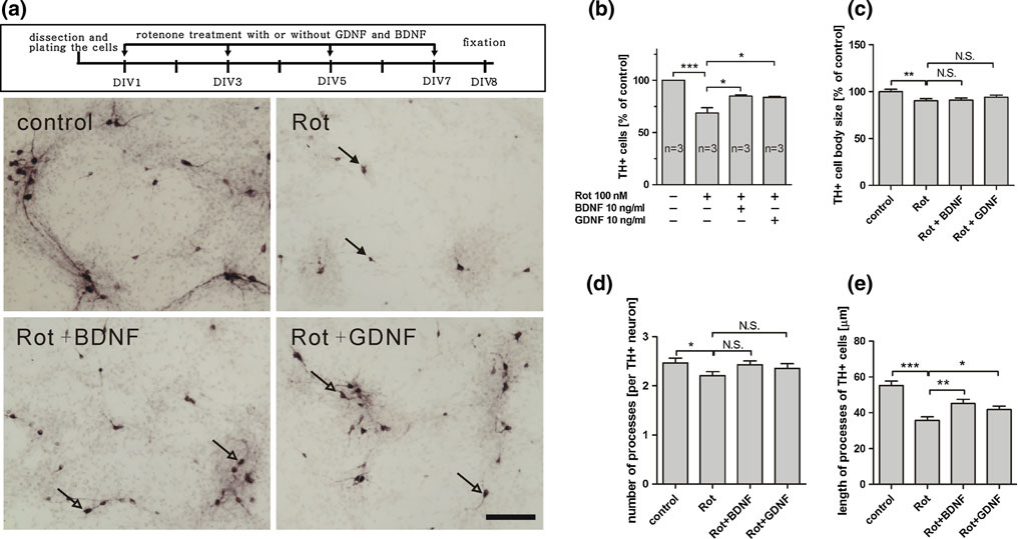
Application of embryonic chicken ventral midbrain culture to evaluate neuroprotection in a rotenone-induced toxicity model. Embryonic chicken ventral midbrain cultures were maintained in serum-free conditions from DIV1 to DIV5 and then treated with rotenone (100 nM) with or without GDNF or BDNF (10 ng/ml) for 2 days (DIV5–7). On DIV8, cells were fixed and stained for TH. **a** Representative pictures of the different treatments show that 100 nM rotenone remarkably reduces the number of TH+ cells, which was significantly rescued by both GDNF and BDNF. Dying and healthy TH+ neurons are depicted by *black* and *white arrows*, respectively. **b**–**e** Quantification of the number, cell body size, number of processes, and length of processes of the TH+ cells in all conditions. All pictures were taken under the same magnification and the scale indicates 100 μm. Data from **b** to **e** are presented as mean ± SEM. *p* values derived from Student’s *t* test (number of TH+ cells, cell body size and length of processes) and Mann–Whitney *U* test (number of processes) are *p < 0.05, **p < 0.01, ***p < 0.001, *NS* not significant. All the experiments were repeated at least three times

To confirm the neuroprotective effect of BDNF and GDNF in our chicken model, we examined the effects of these neurotrophic factors against rotenone, another complex I inhibitor, widely used as a toxic inducer of PD, although not selective for mesencephalic DA neurons. Primary cells were cultured for 5 days in serum-free conditions before the addition of rotenone for 2 days, with or without BDNF and GDNF. We observed a significant decrease in both the number of TH+ neurons and the length of the TH+ cell processes (Fig. 7). This neurotoxic effect was significantly reduced by the treatment with BDNF and GDNF.

These results support that chicken primary culture from embryonic midbrain is a suitable system for studying neuroprotection also in a toxicity condition, as an in vitro model for Parkinson’s disease.

## Discussion

Numerous growth factors have been identified to promote the survival of DA neurons, in both in vitro and in vivo systems. These growth factors include members of FGF, TGF-β, IGF, CNTF, and interleukin families (Roussa et al. 2009; von Bohlen und Halbach and Unsicker 2009). So far, all available therapies for PD can only mitigate the symptoms but not cure the disease. In this context, the screening of molecules for potential trophic effect on mesencephalic dopaminergic neurons represents one of the most promising approaches for the treatment of PD. In this paper, we have analyzed the use of a chicken primary cell culture system as an in vitro model for studying mechanisms leading to neuroprotection in dopaminergic neurons.

The use of chicken embryos for developmental studies has been long and extensively recognized. Since decades, the easily accessible embryo, low-cost maintenance, and especially the potential to perform experimental modifications during embryonic development were the highlighted advantages of this model in research. After the release of the first draft of the chicken genome (Wallis et al. 2004), the use of chicken as a model system has increased due to the possibility of introducing genetic modifications more precisely targeted in time and space in an in vivo setting. Besides the technical and practical advantages, the chicken model presents an alternative to the use of mammals.

In addition to the study of developmental processes in vivo, the chicken model can also provide a supply of neural progenitors for transplantation experiments (Dotan et al. 2010) and has the potential for screening neurodevelopmental toxicity in vitro. First attempts to evaluate neurotoxins in vitro in chicken systems, used embryonic brain, and retinal cells from chicken (Reinhardt 1993). However, to our knowledge, this is the first attempt to study neuroprotection using primary culture of mesencephalic neurons from chicken embryos.

The distribution of the different DA nuclei in the dopaminergic system in chicken, as well as their projection patterns to the striatum, are remarkably conserved with other vertebrate groups (Yamamoto and Vernier 2011). Similarly, various studies indicate that many of the differentiation mechanisms of DA neurons are conserved (Andersson et al. 2006; Farkas et al. 2003). Based on these observations, the use of chicken primary mesencephalic dopaminergic culture could constitute an easy system for screening molecules involved in neuroprotection of DA neurons.

For our work, we selected three representative growth factors—BDNF, GDNF, and FGF2 from three different growth factors families, all of them well known for their ability to act on the nigrostriatal DA neurons. All three factors are conserved through evolution, with high levels of protein identity between human, rodent, and chicken species. The high degree of conservation of BDNF, not only in structure but also in function has previously been described (Gotz et al. 1992). These authors found that the protein encoded by a teleost fish BDNF gene shows survival activity on embryonic chicken (Gotz et al. 1992). Another example of conservation of activity was the treatment of chick dorsal root ganglion explants with human BDNF, promoting neurite outgrowth (Hyman et al. 1991). Same results have been observed for GDNF and FGF2 (Airaksinen et al. 2006; Sasaki et al. 1999).

Consistent with data from human, rat, and mice origin from several laboratories (Beck et al. 1993; Engele and Bohn 1991; Ferrari et al. 1989; Schaller et al. 2005; Studer et al. 1996; Widmer et al. 2000), treatment with BDNF, GDNF, or FGF2 increased the number of TH+ cells in our primary mesencephalic culture from chicken embryos. Furthermore, it increased the cell body size and the complexity of neurite branching. In addition, treatment with FGF2 increased TGF-β secretion when compared with the conditional mediums from control, BDNF- and GDNF-treated cells, as previously reported for midbrain rat cultures (Krieglstein et al. 1998). This indicates that the survival-promoting effect of FGF2 may also be mediated by neurotrophic factor TGF-β. We observed an increase of TGF-β concentration in FGF2-treated cell medium at an early stage of the culture (DIV3) followed by a moderate increase in the later stages (DIV7). At DIV5, there was a strong increase of TGF-β concentration, but the difference was not statistically significant, probably due to the high variance between experiments at that time point. These results indicate that the general mechanisms of neuroprotection of these factors in the midbrain area are conserved between chicken and mammals, thereby supporting chicken midbrain cell culture as a suitable tool for studying neuroprotection of DA neurons in vitro.

In order to explore applications of the chicken cell midbrain culture as an in vitro toxic cellular model for Parkinson’s disease, we examined the neuroprotective effect of GDNF and BDNF against MPP^+^ and rotenone, both of them known to perturb dopamine homeostasis and induce DA cell death in vivo and in vitro conditions (Ahmadi et al. 2003; Lotharius et al. 1999; Przedborski et al. 2000; Schmidt and Ferger 2001). MPP^+^ is a complex I inhibitor which can be specifically transported inside DA neurons through the dopamine transporter present on their cellular membranes (Przedborski et al. 2000). Rotenone, although not selective for mesencephalic DA neurons is also a complex I inhibitor with strong evidence of decreasing the survival of these neurons (Betarbet et al. 2000; Sakka et al. 2003; Sherer et al. 2003). As expected, treatment with MPP^+^ or rotenone decreased the number of TH+ neurons in chicken midbrain DA neuron culture, thus allowing the possibility to test neuroprotective capabilities of potential trophic factors in vitro. Both BDNF and GDNF significantly rescued DA neurons from MPP^+^- and rotenone-induced neurotoxicity as observed by an increase in the number of TH+ neurons.

In conclusion, we have developed a system for screening of potential neurotrophic factors for dopaminergic neurons based on chicken primary DA culture. This system includes the benefits of using chicken as a model species, including its well-known development, easy availability, and that it does not require the sacrifice of pregnant animals to obtain embryos. One of the main limitations of this study is that it is not a mammalian system. Nevertheless, taking into consideration, the high degree of conservation in neuroprotective mechanisms, it could be considered as an initial platform prior to the use of other mammalian systems.
